# Predictive value of shock index variants on 30-day mortality of trauma patients in helicopter emergency medical services: a nationwide observational retrospective multicenter study

**DOI:** 10.1038/s41598-022-24272-9

**Published:** 2022-11-16

**Authors:** Timo Iirola, Johannes Björkman, Mikael Laaksonen, Jouni Nurmi

**Affiliations:** 1 Emergency Medical Services, Turku University Hospital and University of Turku, Turku, Finland; 2 FinnHEMS Research and Development Unit, Vantaa, Finland; 3 Department of Anaesthesiology and Intensive Care Medicine, The University of Helsinki, Helsinki, Finland; 4 Department of Perioperative Services, Intensive Care Medicine and Pain Management, Turku University Hospital and University of Turku, Turku, Finland; 5 Emergency Medicine and Services, Helsinki University Hospital and University of Helsinki, FinnHEMS 10, Vesikuja 9, 01530 Vantaa, Finland

**Keywords:** Medical research, Outcomes research

## Abstract

The original shock index (SI) has been further developed to increase its prognostic value. We aimed to evaluate the predictive value of different SI variants on 30-day mortality among severely injured trauma patients in pre-hospital critical care settings. Adult trauma patients in the national Helicopter Emergency Medical Services (HEMS) registry were evaluated based on the primary outcome of 30-day mortality. SI, SIA (SI multiplied by age), SI/G (SI divided by Glasgow Coma Scale (GCS)), SIA/G (SI multiplied by age and divided by GCS), and SS (SI divided by oxygen saturation) were calculated based on the first vital signs measured at the time of HEMS contact. The area under the receiver operating curve (AUROC) was calculated for each SI variant. In total 4108 patients were included in the study. The overall 30-day mortality was 13.5%. The SIA/G and SI/G had the highest predictive ability (AUROC 0.884 [95% CI 0.869–0.899] and 0.8000 [95% CI 0.7780–0.8239], respectively). The SIA/G yielded good predictive performance between 30-day survivors and non-survivors in the pre-hospital critical care setting.

## Introduction

Trauma is one of the primary sources of death and disability globally, with a substantial percentage of the injured being young adults^1^. The ability to assess the severity of injuries is essential to guide the provision of optimal early intervention and resource allocation throughout the chain of treatment. A simple-to-use instrument that dispenses with the need for complicated calculations is particularly needed in pre-hospital settings, where available resources are scarce.

The shock index (SI), defined as heart rate divided by systolic blood pressure (mmHg), was originally described by Allgöwer et al. in 1967. SI was developed as a simple tool for the detection of circulatory collapse in hemodynamically unstable patients and was later shown to have predictive value in estimating trauma patient mortality^2–5^. A physiological range of SI is defined as 0.5–0.7, with an SI above 1 indicating uncompensated hemodynamic shock, which is accompanied by increased morbidity and mortality^3–6^.

The SI has been further developed, at the expense of its simplicity, to increase its prognostic value with the addition of age, the GCS, and oxygen saturation to the original equation^7^. Furthermore, the functionality of the SI has been assessed by reversing the model (rSI) on the premise that clinicians typically deem uncompensated circulatory shock as when systolic blood pressure is lower than HR, not the other way around^3,6^. Kimura et al. found the reverse SI model incorporating both age and GCS—defined as the rSI multiplied by GCS and divided by age (rSIG/A)—to be the best predictor of mortality especially in elderly, traumatically injured, emergency department (ED) patient populations^6^.

The use of the SI has become common in risk stratification, particularly in the treatment of critically injured patients. Our study aimed to evaluate the predictive ability of different SI variates for 30-day mortality in a highly selected, traumatically injured, pre-hospital critical-care patient population. We hypothesised that SIA/G (rSIG/A) would be the most accurate SI variate for predicting mortality among pre-hospital critical care patients.

## Methods

### Study design

We performed a retrospective observational nationwide multicenter study based on the national HEMS registry from January 1, 2012 to September 8, 2019. The data were combined with hospital discharge registry data and population registry data. The abilities of different modifications to SI in the prediction of 30-day mortality were compared.

The ethical committee of Helsinki University Hospital approved the study protocol (HUS/3115/2019 §194) and the hospital districts responsible for HEMS in Finland (Oulu University Hospital 200/2019 2.7.2019; Helsinki University Hospital HUS/280/2019 9.7.2019; Turku University Hospital J30/19 4.8.2019; the Hospital District of Lapland 32/2019 22.8.2019; Kuopio University Hospital RPL 102/2019 22.8.2019; and Tampere University Hospital RTL-R19580 2.9.2019) and the Population Register Center (VRK/5613/2019-3 1.11.2019) and the Finnish Institute for Health and Welfare (21.2.2020 Dnro THL/2231/5.05.00/2019) also approved the protocol. The ethical committee of Helsinki University Hospital waived the informed consent. The study did not affect patient treatment and therefore patient consent was not required nor acquired. All methods were performed in accordance with the relevant guidelines and regulations, and the Strengthening the Reporting of Observational Studies in Epidemiology (STROBE) statement was followed in reporting the study^8^.

### Setting

The Finnish HEMS system consists of six units, five of which are physician-staffed. One advanced paramedic-staffed unit is located in the sparsely populated northern part of the country. All units operate both by helicopter and rapid response vehicle depending on weather conditions and distance to the patient. HEMS is part of the publicly funded health care system. The units have joint capability for the treatment of critically injured patients, such as pre-hospital anaesthesia and advanced analgesia, thoracic decompression, pelvic binders, and the administration of blood products.

HEMS units are dispatched by the emergency communication centers according to predefined criteria^9^. The ground ambulance crews can also request HEMS to be dispatched. All units respond to both trauma and non-trauma cases. The Finnish HEMS has been recently described in more detail^9^.

### Participants

We included patients (*N* = 4108) aged 18 years and over encountered by HEMS units in Finland and transported to a university hospital with a primary trauma-related diagnosis. Only patients transported to university hospitals corresponding level-one trauma centers were included to represent a seriously injured population. Patients without a correct personal identification code were excluded. Patients with missing data necessary for calculating some of the SI modifications were excluded from that particular analysis.

### Variables

The primary outcome was mortality at 30 days post-incident. The SI modifications evaluated included SI, SIA (SI multiplied by age), SI/G (SI divided by GCS), SIA/G (SI multiplied by age and divided by GCS), and SS (SI divided by oxygen saturation). The first vital signs measured at the time HEMS encountered the patient were used to calculate the indexes.

### Data sources

The pre-hospital data were collected from the national HEMS database. All HEMS missions in the country are entered into this database by the physician or paramedic responsible for treatment immediately after the mission. Earlier studies have addressed the validity of the database and found a very low proportion of missing data and good reliability^9,10^.

The outcome data were collected from the Population Register Center. For hospital discharge diagnosis, data from the National Hospital Discharge Register were searched. Patient entries to this registry are mandatory for all hospitals in Finland. Data from both registries were matched by personal identification code, issued to all Finnish citizens and foreign citizens residing in Finland on a permanent or temporary basis by the Population Register Centre. ICD-10 based Injury Severity Scores (ICISS) were created using hospital discharge diagnoses^11^.

### Statistical methods

The indexes were calculated for patients with sufficient data. Receiver operator characteristic curves (ROCs) were generated to provide a visual representation of the accuracy of mortality prediction for each SI variant. Areas under receiver operator curve (AUROCs) with 95% confidence intervals were also calculated. The indexes of survivors and non-survivors were compared using Mann–Whitney tests. The data are reported as median (interquartile range) or n (%), as appropriate.

The subgroups of trauma without traumatic brain injury (TBI), isolated TBI, and trauma including TBI were studied. These subgroups were used because a TBI may be associated with the Cushing reflex, characterised by high blood pressure and low heart rate, as opposed to hemorrhagic shock^12^. The subgroups were categorised based on patients’ hospital discharge diagnosis codes (Appendix 1).

The sample size was not based on power calculations and all available data were used.

All analyses were performed using IBM SPSS Statistics 27 (IBM Corporation, Armonk, NY, USA). Figures were prepared using Prism 9 (GraphPad Prism 9, GraphPad Software, San Diego, CA, USA).

## Results

**Figure 1:**
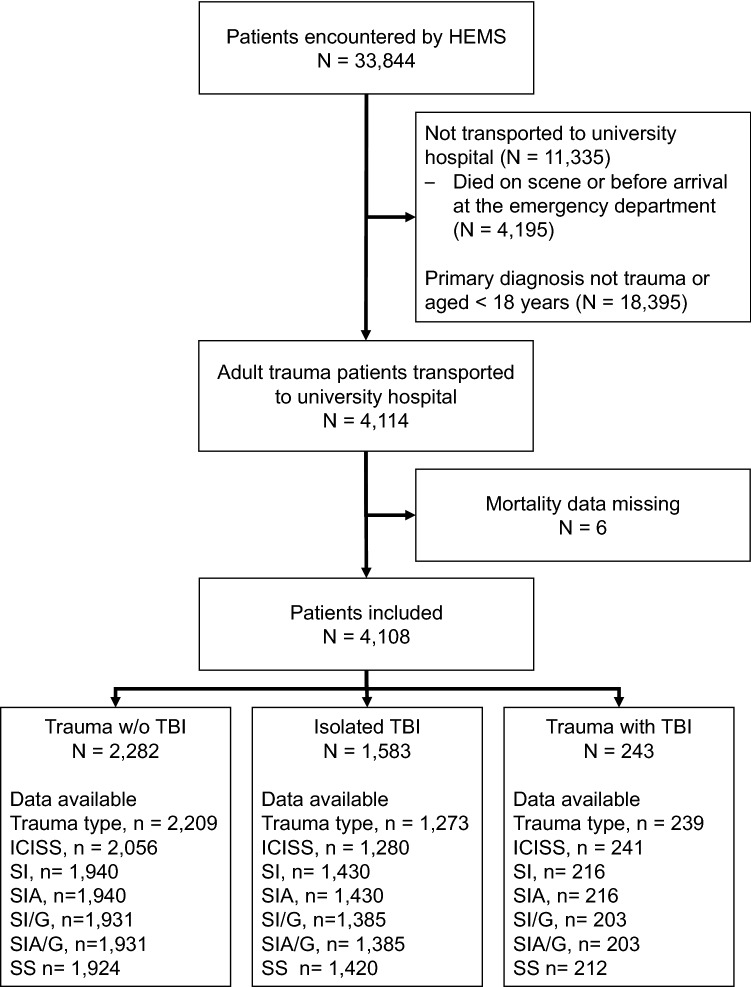
Patient selection flowchart. HEMS, Helicopter Emergency Medical Services; TBI, traumatic brain injury; ICISS, ICD-10 based Injury Severity Score; SI, shock index; SIA, SI multiplied by age; SI/G, SI divided by Glasgow Coma Score; SIA/G, SI multiplied by age and divided by Glasgow Coma Score; SS, SI divided by oxygen saturation.

During the study period, the HEMS units encountered 4108 patients who met the inclusion criteria (Fig. 1). This sample consisted of 2282 cases of trauma without TBI (55.6%), 1583 isolated TBI (38.5%), and 243 trauma with TBI (5.9%). Six patients had extremely compromised hemodynamics–four with blood pressure < 50 mmHg and two with a heart rate of < 30 beats per minute. The patient characteristics are described in Table 1.

**Table 1:** Patient characteristics.

	All patients (n = 4108)		Trauma without TBI (n = 2282)		Isolated TBI (n = 1583)		Trauma with TBI (n = 243)	
	Median/n	IQR/%	Median/n	IQR/%	Median/n	IQR/%	Median/n	IQR/%
Age, years	49	31–65	44	28–59	57	36–71	47	32–59
Sex, male	3027	74	1728	76	1112	70	187	77
Type of injury, blunt	3408	92	1944	88	1233	97	231	97
ICISS	0.91	0.79–0.98	0.94	0.88–0.98	0.83	0.69–0.96	0.73	0.60–0.84
**First vital signs by HEMS**								
Heart rate, min^−1^	89	75–102	90	76–102	87	72–101	90	76–110
Systolic blood pressure, mmHg	130	112–150	126	107–142	140	120–164	130	111–151
Oxygen saturation, %	97	94–99	97	94–99	97	94–99	98	93–99
Glasgow Coma Score	13	5–15	15	14–15	6	3–11	9	4–13
**Pre-hospital critical care interventions**								
Advanced airway management	1120	27	246	11	753	48	121	50
Vasoactive drugs	688	17	188	8	428	27	72	30
**Time intervals**								
From alarm to HEMS, min	24	17–38	25	17–37	24	16–38	27	17–39
On-scene time, min	20	12–32	17	9–28	24	15–34	25	17–39
Transport time, min	29	17–42	29	18–42	28	16–42	30	19–43

TBI, Traumatic brain injury; IQR, interquartile range; ICISS, ICD-10 based Injury Severity Score; HEMS, Helicopter Emergency Medical Services. Pre-hospital critical care intervention means an intervention that has been performed during the prehospital period by the HEMS unit. Therefore, they have not affected the vital signs used for calculation of the shock index variants.

The overall 30-day mortality rate was 13.5% (553/4108). The mortality rates in the subgroups were 4.5% (103/2282) for trauma without TBI, 14.0% (34/243) for trauma with TBI, and 26.3% (416/1583) for isolated TBI. A comparison of the characteristics of the SI modifications between survivors and non-survivors is presented in Table 2.

**Table 2:** Comparison of different shock index variations between 30-day survivors and non-survivors. Data are expressed as median (interquartile range).

	Survivors (N = 3 050)		Non-survivors (N = 469)		*P* value
SI	0.67	(0.55–0.81)	0.57	(0.44–0.78)	< 0.001*
SIA	30	(21–39)	38	(28–53)	< 0.001*
SI/G	0.0513	(0.041–0.074)	0.129	(0.079–0.206)	< 0.001*
SIA/G	2.34	(1.57–3.57)	8.05	(4.96–12.4)	< 0.001*
SS	0.0068	(0.0056–0.0084)	0.0058	(0.0044–0.0080)	< 0.001*

*Mann–Whitney. SI, shock index; SIA, SI multiplied by age; SI/G, SI divided by Glasgow Coma Score; SIA/G, SI multiplied by age and divided by Glasgow Coma Score; SS, SI divided by oxygen saturation.

**Figure 2:**
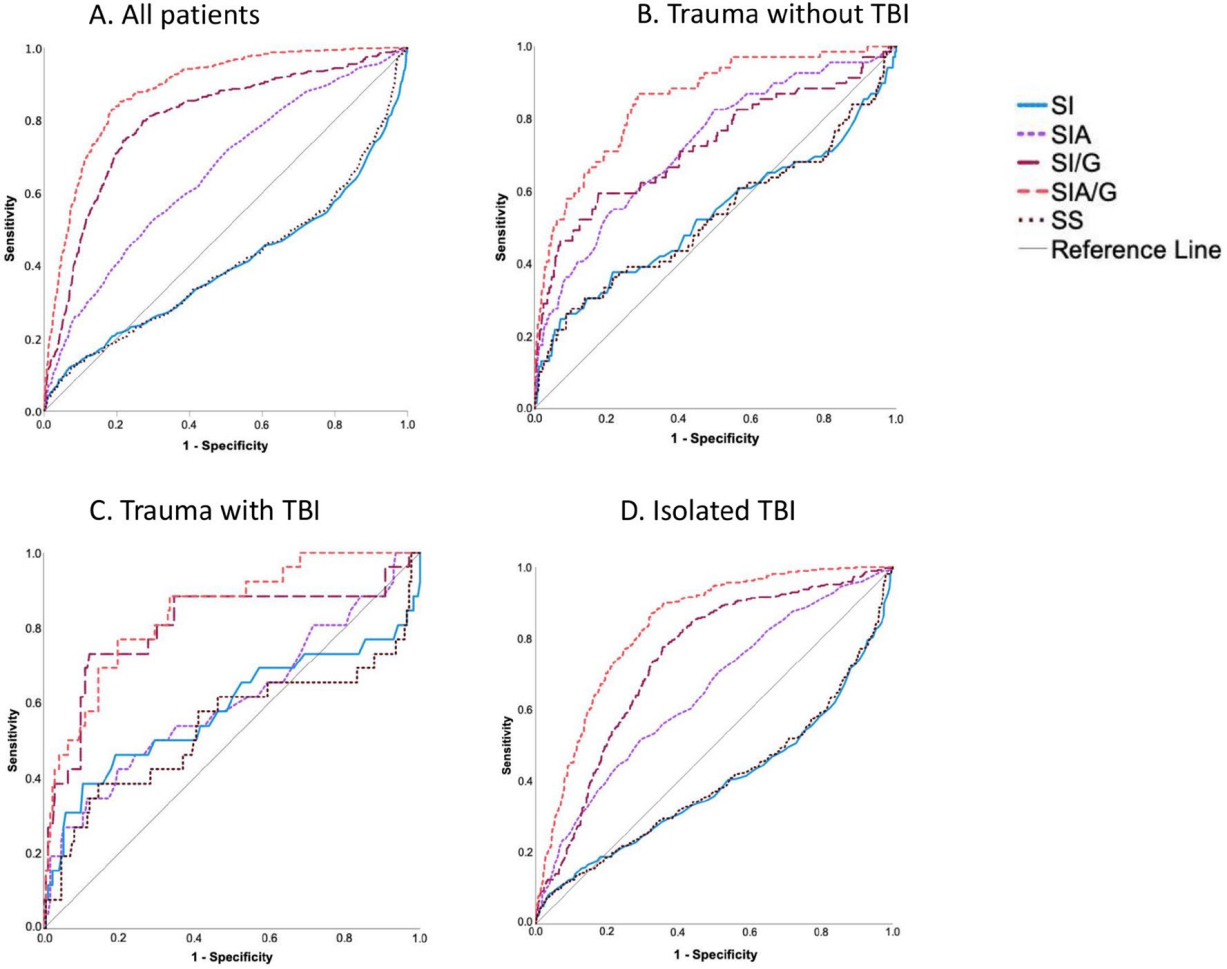
Receiver operating characteristic curves for the different shock index modifications for all patients and subgroups. The number of patients varies depending on the amount of missing data needed to calculate specific indexes. TBI, traumatic brain injury; SI, shock index; SIA, SI multiplied by age; SI/G, SI divided by Glasgow Coma Score; SIA/G, SI multiplied by age and divided by Glasgow Coma Score; SS, SI divided by oxygen saturation.

**Figure 3:**
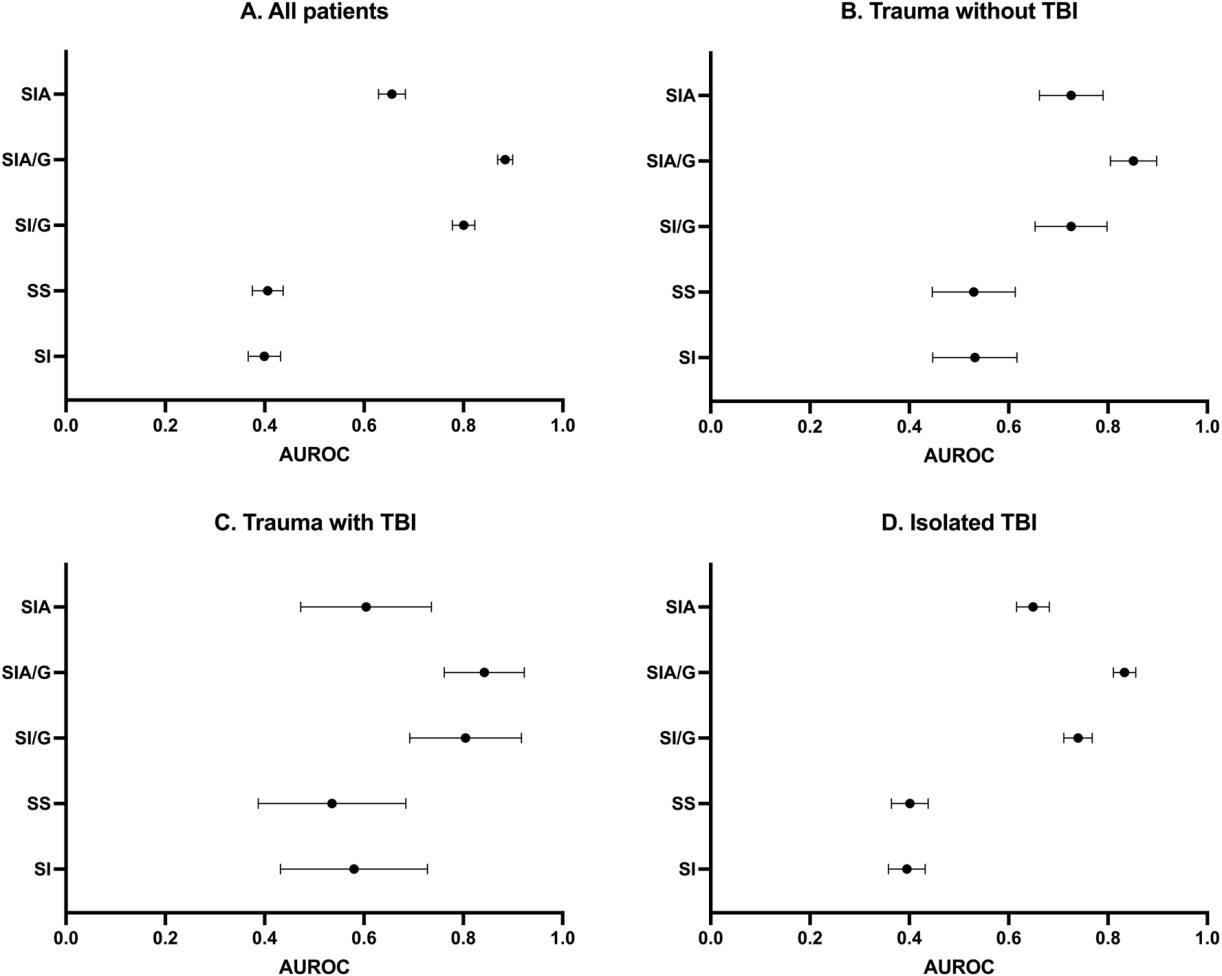
(**A–D**) Area under receiver operator curve (AUROC) of different variations of shock index in all trauma patients, trauma patients without traumatic brain injury, trauma patients with traumatic brain injury, and patients with isolated brain injury. Lines represent 95% confidence intervals. The number of patients varies depending on the amount of missing data needed to calculate specific indexes. TBI, traumatic brain injury; SI, shock index; SS, SI divided by oxygen saturation; SI/G, SI divided by Glasgow Coma Score; SIA/G, SI multiplied by age and divided by Glasgow Coma Score; SIA, SI multiplied by age.

The SI/G and SIA/G indexes provided the highest predictive ability. The ROC curves of the different SI modifications for all patients and subgroups are presented in Fig. 2A–D. The AUROC results and 95% confidence intervals of the different SI modifications by subgroup are presented in Fig. 3.

**Figure 4:**
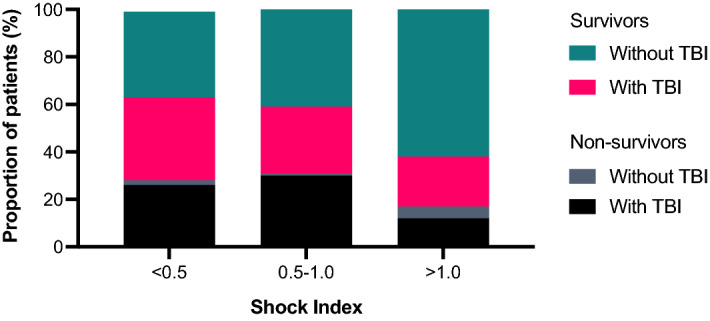
Proportions of survivors and non-survivors with and without traumatic brain injury (TBI) in low (< 0.5), normal (0.5–1.0), and high (> 1.0) shock index categories.

Figure 4 shows the proportions of survivors and non-survivors in patients with or without TBI with a low, normal, or high SI. A high number of non-surviving TBI patients was observed in the low or normal SI categories. As opposite, the majority of non-survivors without TBI who were characterised by a high SI.

## Discussion

The results show that the SI derivative SIA/G was superior to the other SI variants studied in predicting mortality among a selected, pre-hospital critical-care trauma-patient population. The same findings were also observed as a result of the analysis of the subgroups. Hence, the addition of age and GCS increased the predictive ability of the SI significantly.

The identification of patients requiring pre-hospital critical care is of paramount importance to dispatch the proper resources and allow for appropriate advance preparation at the receiving ED. The SI and its derivatives are useful for anticipating the need for the highest-level trauma-team activation and blood transfusions, as well as the need for mechanical ventilation^3–6,13–16^. Therefore, as a simple tool, the SI can also be considered of considerable potential value to the pre-hospital setting, both as a tool for HEMS dispatch and cancellation as well as resource allocation.

The GCS, after its conception in the 1970s, has been widely adopted throughout the world to describe patients’ level of consciousness. The GCS has been shown to be independently and strongly correlated with mortality for patients with TBI^17,18^. Age, as an independent variable, has been shown to be a considerable factor affecting mortality in severely injured patients, with higher age corresponding to elevated mortality^4,5^.

In a large, retrospective study by Kimura et al., based on vital signs registered on admission at emergency department of 168,517 patients extracted from the Japan Trauma Data Bank, the SI derivate rSIG/A was the most accurate in predicting trauma patient mortality^6^. The trauma patients in the pre-hospital critical care setting in our study represent a highly selected group of the most severely injured patients. This could account for why the mortality rate in the present study was twice as high as that found by Kimura (13.5 vs. 6.4%).

Furthermore, the proportion of patients with TBI was markedly high in our study. The high incidence of TBI most likely explains the poor performance of the original SI in our cohort. Despite the differences in the time of the vital sign measurement and the type of the patient cohort, our results confirm the superiority of SIA/G to other SI measures.

In the subgroup analyses, the confidence intervals were relatively wide among patients with trauma and traumatic brain injury. SIA/G was still the best shock index, even though the 95% confidence intervals overlapped. In our opinion, the wide dispersion is at least partly due to the small number of patients in that subgroup.

The current study sampled a highly select population of trauma patients in Finland, which has a high-quality health care system, a relatively high amount of TBI, and a relatively low incidence of penetrating trauma. Consequently, this aspect must be considered when generalising the results.

### Strengths and limitations

The national HEMS registry comprises all HEMS missions within Finland, which represents a considerable strength of the present study. Furthermore, the use of patients’ personal identification numbers allowed registries to be combined, which resulted in a very low proportion of missing follow-up data.

Despite its merits, our study is subject to several limitations. First, in Finland, HEMS is dispatched only to selected incidents, HEMS unit may not have been able to attend every call, and the mission may have been canceled or turned down by a HEMS clinician. Therefore, although the current study purposefully reported on a highly select trauma-patient cohort, participant selection was somewhat unsystematic. Second, even though the treatment of trauma patients is likely to be relatively consistent in Finland, there is a lack of national treatment protocols, which may have affected the results. Third, the data sourced from the above-mentioned registries are unvalidated. Fourth, the time of measurement of the vital signs used in calculation of the shock index variants was upon arrival of the HEMS unit. Therefore, the vital signs may have been artificially enhanced by potential first-line, life-saving treatments provided by EMS units prior to HEMS arrival. Finally, the high mortality rate and high proportion of patients with TBI may limit generalising the results.

## Conclusions/summary

This study demonstrates that the addition of age and GCS signicantly increases the predictive capability of the SI to anticipate 30-day mortality for trauma patients in pre-hspital critical care setting This findings is most likely explained by patient selection resulting in a high number of TBI patients—with a markedly high mortality rate.

## Supplementary Information

Supplementary Information.

## Data Availability

Anonymized dataset is available upon reasonable request and may be obtained by contacting the corresponding author.
